# Dr. Walker, on Mr. Goldson's Pamphlet

**Published:** 1804-08-01

**Authors:** John Walker


### *To the Editors of the Medical and Physical Journal.*

Gentlemen, *Salisbury Square,* 13, *vij.* 1804.

WILL ye have the ingenuousness, the candour, to insert in your next Number, a most sincerely meant censure upon yourselves? While the sentiment of Horace, *Sunt delicta tamen, quibus ignovisse velimus,* may on this occasion be generally the feeling of your readers; I confess for myself, that on your treatment of the great subject of Vacciolation, *Indignor quandoque bonus dormitat Homerus.* How could ye, in your Review of Goldson's pamphlet, think of of taking up the time of your readers in considering what were the merits or demerits of the medical men in any particular neighbourhood remote from, or near to, 'Head quarters of science!' Whichsoever seat of the Muses be the head quarters of medical science in the British empire, mails, stage-coaches, &c. are the never failing *aids du camp* which convey the '*ordres du jour* throughout, and beyond the whole imperial field! But, chiefly, on the subject which forms the *title* of the pamphlet, I have never seen you so flat; and, pray, what can ye have meant by the entire pamphlet claiming "an attentive perusal from all the partisans, friends and well-wishers of Dr. Jenner's discovery?"

Ye have promised yourselves too much, if ye have expected that the pernicious pamphlet would hereby become extinct; but, I correct myself: It occurs to me, that ye may have thought it ought to be in the hands of every friend of vacciolation, that they might always have it in their power to shew the fallacy of the author's conclusions, the incompleteness of his statements. In the consciousness of his own unwavering veracity, he seems quite to have forgotten that the statement of medical cases requires judgment as well as truth, and that the proof of the concurrence of both in the report is always due to the reader, who may have perhaps never before heard the name of the author, and who owes no credit to his *ipse dixit.*

Since my last, it has occurred to me, that I may myself have appeared somewhat deficient in not offering any authority for the statement of the pamphlet being gratuitously distributed. I could not, without a sort of breach of confidence, unveil the springs whence the streams have already borne it away so far and wide throughout the empire; but I may give some idea of the activity and extent of the diffusing influence, by the mention of the arrival from Dublin of a professional friend, who received it a few days before his leaving Ireland, without note or message, in an *envelope* with the following address: James Rivers, Esq. 26, Queen Street, or at the Hospital of the House of Industry, Brunswick Street.

Pythagoras, in the fulness of his joy, is said to have sacrificed a hecatomb to the Muses, on his discovery of the proposition which forms the 47th of Euclid. How happens it that our author, on his supposed discovery so shockingly serious, possesses an apathy more than philosophic? I once saw the captain of a Guineaman drink glass after glass of brandy, till he fell asleep on his chair. "There is is a man, said one of the company—but the tear of compassion never moistened his cheek." Where were all the sympathies, the ordinary feelings of humanity, when our discoverer should have revealed to the world, that the Jennerian inoculation might not ultimately prove a permanent prophylactic; that the Small Pox, at some future period, might become a greater scourge to the world than ever? Does any expression of regret or sorrow escape him on the melancholy occasion? With all his anticipation of a *Vox in Rhama, lamentatio, et fletus, et ejulatus multus*—the Rachels *plorantes suos filios* seem neither to have excited his sighs nor tears. Like the geometricians and chemists of Burke, 'bringing from the dry bones of their diagrams, and the soot of their furnaces, dispositions that make them worse than indifferent about those feelings and habitudes, which are the supports of the moral world,' he is too much absorbed in his favourite object. He seemeth a Gallio, not to care for these things. But let us hope he only seemeth. He wishes not to provoke controversy; and he has provoked it. He *only* asks for further investigation; and further investigation is not wanted. Let him inform himself of what has been already done. I hope he will shortly be fully convinced that he has hitherto only been 'puzzled in mazes, and perplext with errors;' when a most respectable, a truly honourable, duty may fall to his lot, that of making public acknowledgement. This is the conduct I took upon me to recommend more than ten years ago to the Yatton demoniac, from whom about seven devils should have been exorcised, by perhaps about as many fanatical preachers at Bristol; but in this case the confessor may neither have to say *peccavi* nor *poenitui*, but simply, 'whereas I was blind, now I see.'

Having taken the liberty of introducing the grave subject of moral habitudes and feelings, and even of insinuating an inattention to them on the part of our author, let me beg of him to excuse this greatest possible breach of liberality. It is a very easy thing to make fine professions of humanity. I have learnt to set very little by them, from hearing so much of them amid the revolutionary storms of the fickle nation, where I had only to turn my eyes to a public building, to be disgusted with the discordant inscriptions "*Humanité, Fraternité, &c. ou la mort.*" Let us hope then that William Goldson, with every appearance of apathy amidst his death-threatening alarms, may yet feel as a man. As such I would wish to congratulate him on his own total failure. Yet a little, and he may may have to declare to the world, I am happy, supremely happy, in being at length fully convinced that all my conjectures on the inefficacy of the Jennerian Inoculation were erroneous.

> "The grateful nations one loud pœan raise;
> A wond'ring world resounds our JENNER's praise.

18, vii, 1804. Will the extreme importance of the subject (Vacciolation) on which I have ventured to commit myself before the medical world, through the medium of your Journal, induce you to receive yet a few words more, probably the last I shall now have to offer? They are excited by, or rather extracted from, the "Answer to Mr. Goldson, by John Ring, member of the Royal College of Surgeons." I can neither adopt the *nunc dimittis* of Simeon in the Temple, nor the last dying words of Wolf on the heights of Abraham, on this occasion; but my joy is great. The estimable author, in the consciousness of having done his duty, of not having lived in vain, may derive something of this last consolation of the human heart. He has furnished 'an antidote to the poison.' How completely he has done it, ye will no doubt have to shew in criticising the work; but do give me leave to point out to your anxious readers (Medical and Physical Journal, page 553, June, 1804) how little dependance they ought to place on the accuracy of the statements which may have excited their alarms.

Page 7. "As Mr. Goldson assures us he does not wish to spread vain alarms, and that he should not think himself justifiable in concealing the cases which have fallen under his observation; it is difficult to conceive how he could think himself justifiable in bringing forward a partial statement of a case, and concealing a circumstance, which alone is sufficient, in this instance, to vindicate the character of vaccination from the charge he has brought against it."

"It must be recollected, that if Mr. Goldson is not accurate in his statements, if the fidelity of his narrations can be impeached, whether the fault be a treacherous memory, or too many other avocations, his whole testimony must fall to the ground."

Page 8. "Mr. Goldson is an active magistrate, and a surgeon in considerable practice. It is therefore more liberal to suppose, that other avocations prevented him from attending to the case like an affectionate parent; than that he would have suppressed any important facts, in order to make it appear, &c."

Let

Let me now conclude by giving you the address of a few more applicants from the southward, for matter at the Central House; but, without any comment, lest William Goldson should again discover many wilful misrepresentations, as he asserts, but fails to prove, while he entirely forgets to attempt to defend all his incautious or unguarded statements, against the unmannerly remarks of

Yours, sincerely,

JOHN WALKER.

"Lord Seaforth, Governor of Barbadoes (Portsmouth, on his way to the West Indies)
Mr. Tristram Harper, Gosport.
Mr. Tupper, on his way to Guernsey.
Mr. F. Weber, Hilsea Barracks, Surgeon to the King's German Legion.
Mr. Mill, Hilsea Barracks, Ensign of the 1st. West India Regiment.
Edward Jackson, Guildford.
Morris Birkbeck, Wanbro'.
Mrs. Lewin, Ridgeway Castle, Southampton.
Lord Egremont.
T. Hodson, Lewes.
C. Morgan, Henfield.
The Widow of Don Manuel Azlor, Viceroy of Navarra.
The Count de Bureta, of Saragosa.
Mr. Long, Hailsham."

We have no objection to shewing our candour by the insertion of Dr. Walker's reproof; and we entertain no doubt of his perfect sincerity in the great cause of humanity. But surely he would not wish us to set ourselves up as Dictators. We shall always wish our Journal to be a 'Ulysses's bow, on which every man of genius and learning may try his strength and skill.' If any man fail in his attempt to bend it, he only exposes the impotence of his arm; his shafts fall harmless. And the giving publicity to such failure, we consider as one of the most important services that we can render to science and to humanity. EDIT.

---

